# The Dark Triad and Deception Perceptions

**DOI:** 10.3389/fpsyg.2019.01811

**Published:** 2019-08-13

**Authors:** Benno Gerrit Wissing, Marc-André Reinhard

**Affiliations:** ^1^Department of Psychology, University of Kassel, Kassel, Germany; ^2^Department of Psychology, Social Psychology, University of Kassel, Kassel, Germany

**Keywords:** dark triad, pid-5, deception, deception detection, deception production

## Abstract

The present cross-sectional study (*N* = 205) tested the hypothesis that the Dark Triad traits – narcissism, Machiavellianism, and psychopathy – and the PID-5 maladaptive personality traits – Negative Affectivity, Detachment, Antagonism, Disinhibition, and Psychoticism – are associated with specific deception-related perceptions: perceived cue-based deception detectability, perceived deception production ability, and perceived deception detection ability. Participants completed personality and deception measures in an online setting. All three Dark Triad traits and Antagonism were associated with perceived deception production ability, but not (substantially) with perceived deception detection ability and cue-based deception detectability. The results provide a more fine-grained picture of biases associated with the Dark Triad traits in the context of deception and further support the relevance of Antagonism and Detachment as deception-relevant personality traits.

## Introduction

Research on the Dark Triad (Paulhus and Williams, 2002) – the moderately associated personality traits of narcissism, Machiavellianism, and psychopathy – has accrued in the last 17 years. Viewed through the lens of the two main personality frameworks, the Five-Factor Model (FFM; Costa and McCrae, 1992) and the HEXACO model (Lee and Ashton, 2005), the three traits are constituted primarily by low levels of agreeableness (Paulhus and Williams, 2002) and low Honesty-Humility (Lee and Ashton, 2005). Given that agreeableness can be defined as the willingness to cooperate, a low manifestation of this trait can have negative consequences for group inclusion (Buss, 1991). Accordingly, individuals high in Dark Triad traits tend to be lower in communion and more agentic (Jonason et al., 2010; Jones and Paulhus, 2010) and Machiavellianism and narcissism are linked with higher independent self-construals (Jonason et al., 2017). Beyond their shared variance, the Dark Triad traits also exhibit substantial distinctiveness: Narcissism is defined by entitlement, grandiosity, dominance, and superiority (Raskin and Hall, 1979; Corry et al., 2008). Machiavellianism is characterized by an amoral, cold, and cynical view of the world and strategic manipulativeness (Christie and Geis, 1970). Psychopathy is associated with higher risk-taking, impulsivity, lower empathy, and lower neuroticism (Hare, 1985). Some researchers have argued for domain-specific adaptiveness of the Dark Triad traits (see Jonason et al., 2014a).

According to a foundational meta-analysis, humans are slightly better than chance at detecting deception (Bond and DePaulo, 2006). Moreover, humans tend to be truth-biased in their response, that is, they assume that others are truthful independently of their actual truth status (Levine et al., 1999) and more variance is found in response bias than in deception detection accuracy (Bond and DePaulo, 2008; Schindler and Reinhard, 2015). The almost chance converging deception detection performance might be explained by the low availability of behavioral cues to deception (Hartwig and Bond, 2011).

Low agreeableness and low Honesty-Humility – the primary correlates of the Dark Triad traits within well-established personality frameworks – are both associated with deceptive behavior. Correspondingly, one attempt to capture the personality-theoretical core of the three traits has converged on manipulation-callousness (Jones and Figueredo, 2013) – thus, further highlighting the close connection between the Dark Triad traits and deception. There is evidence that especially Machiavellianism and psychopathy are associated with interpersonal deception production frequency (Kashy and DePaulo, 1996; Baughman et al., 2014; Jonason et al., 2014b) and the former additionally with amplitude (high-stakes deception; Azizli et al., 2016). On the contrary, narcissism is associated with intrapersonal deception, that is, self-deception (for example, Paulhus and Williams, 2002). Three contextual variables have been identified that influence deceptive behavior exhibited by the Dark Triad traits: risk level, ego depletion, and deception target (self vs. others) (Jones and Paulhus, 2017). A study which measured the Dark Triad traits and the levels of testosterone and cortisol before and after a deception production task (video-taped lying) found a physiological response pattern – cortisol decrease for men high in Machiavellianism (and suggestively so for narcissism and psychopathy as well) and testosterone increase post-test for narcissism and psychopathy – in line with the hypothesis that the Dark Triad traits represent an evolved “cheater strategy” (Dane et al., 2018).

Research on the relationship between deception detection accuracy and personality traits is limited (Aamodt and Custer, 2006). The data in the existing literature on dark personality traits and deception detection ability are mixed. For example, primary psychopathy is associated with lie detection accuracy in men (Lyons et al., 2013), while other research did not report such an association (for example, Peace and Sinclair, 2012). Additionally, no superior lie detection accuracy for individuals high in Machiavellianism was reported (Zuckerman et al., 1981), except for one study that reported woman high in Machiavellianism displayed a higher lie detection ability (Lyons et al., 2017). Moreover, in an interactive deception task, no correlation between deception detection or deception production ability and the Dark Triad traits was detected (Wright et al., 2015). Parallel results regarding deception detection ability were reported (Wissing and Reinhard, 2017). This line of research corresponds to the result pattern of previous research (Bond and DePaulo, 2006, 2008) and expands it to the Dark Triad traits, indicating that these traits are not substantially associated with actual deception detection or deception production ability, but with varying amplitudes and frequencies of deception production and various deception-related judgmental and perceptual biases.

In sum, previous research suggests that self-perceived deception production abilities (Giammarco et al., 2013) and multiple deception-related biases (Wissing and Reinhard, 2017) are associated with the Dark Triad traits. Based on these findings, this study tried to replicate the association of Dark Triad traits with self-perceived deception production abilities and additionally investigated if the Dark Triad personality traits are also correlated with perceptions potentially relevant to the process of deception detection. In detail and beyond the replication regarding self-perceived deception production abilities, this study assumed that all three Dark Triad traits – narcissism, Machiavellianism, and psychopathy – are positively correlated with perceived cue-based deception detectability and self-perceived deception detection abilities. Based on the finding that the Big Five trait Agreeableness is moderately negatively correlated with perceived ability to deceive (Giammarco et al., 2013), it was assumed that the corresponding dimension in PID-5 maladaptive personality space – Antagonism – is positively correlated with self-perceived deception detection abilities. The PID-5 maladaptive personality traits were also included instead of the Big Five, because research found that the PID-5 traits outperform the Big Five as predictors of the Dark Triad traits (Grigoras and Wille, 2017).

## Materials and Methods

The study was conducted in full accordance with the Ethical Guidelines of the German Association of Psychologists (DGPs) and the American Psychological Association (APA). Moreover, by the time the data were acquired, it was also not required at Kassel University, nor at most other German universities to seek ethics approval for simple studies on personality and attitudes. Thus, ethics approval was not required as per applicable institutional and national guidelines and regulations. The study exclusively makes use of anonymous questionnaires. No identifying information was obtained from participants. The participants were explicitly informed that the data are treated confidentially. The informed consent of the participants was provided online: every participant had to agree to the following statements: “I understand that my participation is voluntary and that I may withdraw from the study at any time without explanation” and “I hereby confirm that I am at least 18 years old, and that I agree to take part in this study.” Furthermore, they could withdraw from the study at any time.

## Statistical Power and Participants

The correlations between the Dark Triad traits were estimated based on the average effect sizes (*r*) for the intercorrelations between the three traits reported in a meta-analysis (*r*s = 0.34, 0.38, 0.58; Muris et al., 2017). The correlations between the three Dark Triad traits and cue-based deception detectability and perceived deception detection ability were estimated as *r* = 0.25. The *r* = 0.25 was selected based on an assumed weaker correlation of the Dark Triad traits with cue-based deception detectability and perceived deception detection ability vs. perceived deception production ability (*r*’s = 0.33–0.41; Giammarco et al., 2013). These values were entered into the statistical power analysis tool G*Power (Faul et al., 2009) and the required minimum sample size *N* = 157 for a linear multiple regression with *k* = 3 predictors at alpha level *α* = 0.05 with Power 1 − *β* = 0.95 for an estimated small to medium effect size *f*^2^ = 0.11 (calculated via *p*^2^) was calculated. For a simple correlation test of *r* = 0.25 (two-tailed) at alpha level *α* = 0.05 with Power 1 − *β* = 0.95, the required sample size was *N* = 202.

Participants were recruited with Amazon’s Mechanical Turk (MTurk), selecting exclusively MTurk Masters (a high-performance group that demonstrated accuracy in the past per Amazon), and were paid a small fee (1$). *n* = 17 participants dropped out before the first dependent variable measurement was finished, resulting in the final sample (*N* = 205; 58.54% male, 41.46% female; *M*_age_ = 37.76, SD = 10.80, age range: 22–70 years; 94.8% native English speaker, 93.66% living in the United States of America, 73.66% Caucasian; 72.68% employees).

## Procedure and Measures

The Dark Triad traits were measured with the Short Dark Triad (SD3; Jones and Paulhus, 2014), a 27-item short self-report instrument that measures narcissism (for example, “People see me as a natural leader.”; *α* = 0.87), Machiavellianism (for example, “I like to use clever manipulation to get my way.”; *α* = 0.87), and psychopathy (for example, “People who mess with me always regret it.”; *α* = 0.81) with nine items each on a 5-point Likert-type scale (1 = *disagree strongly*, 5 = *agree strongly*).

The Personality Inventory for DSM-5 Brief Form (PID-5-BF; Krueger et al., 2012; American Psychiatric Association, 2013) was used to measure the five maladaptive personality trait domains of the PID-5 model with 25 items in total, consisting of Negative Affectivity (for example, “I worry about almost everything”; *α* = 0.82), Detachment (for example, “I often feel like nothing I do really matters”; *α* = 0.83), Antagonism (for example, “It’s no big deal if I hurt other peoples’ feelings”; *α* = 0.83), Disinhibition (for example, “People would describe me as reckless”; *α* = 0.88), and Psychoticism (for example, “My thoughts often don’t make sense to others”; *α* = 0.85) using a 4-point Likert-type scale (0 = *very false/often false*, 3 = *very true/often true*).

Participants read that the next questions would be about their thoughts on how people behave when they are lying or telling the truth. Cue-based deception detectability was measured using 22 statements based on previously identified beliefs about cues of deception (Hartwig and Bond, 2011) referring to verbal (three items; for example, “Deceptive statements are less detailed than truthful statements”), para-verbal (four items; for example, “Liars pause less than truth tellers”), and non-verbal aspects (15 items; “Liars blink less than truth tellers”) of deceptive behavior. A 7-point Likert-type scale ranging from −3 (for example, “Deceptive statements are less detailed than truthful statements”) *via* 0 (= *no difference*) to +3 (for example, “Deceptive statements are more detailed than truthful statements”) was used. Negative values were recoded to positive ones (−1 = 1, −2 = 2, −3 = 3), resulting in the final scale [value range: (0, 3)]^1^. (Note 1) For each statement, participants should select the answer that was most closely aligned with their opinion on the given statement. Exploratory factor analysis using maximum likelihood suggested a one-factor structure as being sufficient with factor loadings between 0.42 and 0.79 (40% explained variance). One item with factor loading = 0.42 < 0.50 was removed, resulting in the final scale with 21 items. Exploratory factor analysis on the final scale using maximum likelihood suggested a one-factor structure as being sufficient with factor loadings between 0.55 and 0.80 (41% explained variance) (*α* = 0.93, *ω_h_* = 0.76, *ω_t_* = 0.95).

Using a 7-point Likert-type scale (1 = *strongly disagree*, 7 = *strongly agree*) perceived deception detection ability was measured with six items (for example, “In general, liars can be easily recognized,” “In general, I’m good at detecting liars”; *α* = 0.85). Exploratory factor analysis using maximum likelihood suggested a one-factor structure as being sufficient with factor loadings between 0.36 and 0.96 (51% explained variance). One item with factor loading = 0.36 < 0.50 was removed, resulting in the final scale with five items. Exploratory factor analysis on the final scale using maximum likelihood suggested a one-factor structure as being sufficient with factor loadings between 0.63 and 0.98 (58% explained variance) (*α* = 0.86, *ω_h_* = 0.72, *ω_t_* = 0.93).

Using a 7-point Likert-type scale (1 = *strongly disagree*, 7 = *strongly agree*) perceived deception production ability was assessed with three items (for example, “I’m a good liar”; *α* = 0.77, *ω_t_* = 0.78). Exploratory factor analysis using maximum likelihood suggested a one-factor structure as being sufficient with factor loadings between 0.71 and 0.76 (53% explained variance).

## Results

Descriptive statistics for all variables can be seen in Table 1.

**Table 1:** Descriptive statistics.

	*M* (SD)		
	Overall (*N* = 205)	Sex: male (*n* = 120)	Sex: female (*n* = 85)
Deception			
Cue-based deception detectability	1.42 (0.65)	1.42 (0.57)	1.42 (0.75)
Perceived deception detection ability	4.40 (1.16)	4.54 (1.05)	4.20 (1.28)
Perceived deception production ability	3.64 (1.36)	3.83 (1.36)	3.39 (1.33)
Dark Triad			
Narcissism	2.54 (0.81)	2.77 (0.85)	2.22 (0.63)
Machiavellianism	3.00 (0.79)	3.19 (0.83)	2.74 (0.65)
Psychopathy	2.12 (0.73)	2.31 (0.72)	1.84 (0.65)
PID-5			
Negative affectivity	0.94 (0.71)	0.85 (0.68)	1.06 (0.74)
Detachment	0.88 (0.75)	0.92 (0.76)	0.83 (0.73)
Antagonism	0.61 (0.61)	0.72 (0.65)	0.47 (0.54)
Disinhibition	0.54 (0.64)	0.57 (0.62)	0.49 (0.66)
Psychoticism	0.57 (0.63)	0.61 (0.58)	0.52 (0.69)

### Cue-Based Deception Detectability

As seen in Table 2, there was no substantial association pattern between dark and maladaptive personality traits and cue-based deception detectability. Only Detachment showed a suggestive negative association pattern with cue-based deception detectability.

**Table 2:** Zero-order Pearson correlation coefficients with 95% CIs (in brackets) for the Dark Triad traits, the PID-5 traits and the deception variables.

	Cue-based deception detectability	Perceived deception detection ability	Perceived deception production ability
Dark Triad			
Narcissism	−0.01 [−0.15, 0.13]	0.16 [0.02, 0.29]^*^	0.33 [0.21, 0.45]^***^
Machiavellianism	−0.05 [−0.18, 0.09]	0.12 [−0.02, 0.25]	0.45 [0.34, 0.56]^***^
Psychopathy	−0.01 [−0.14, 0.13]	0.14 [0.01, 0.28]^*^	0.44 [0.32, 0.54]^***^
PID-5			
Negative affectivity	0.02 [−0.12, 0.15]	−0.13 [−0.26, 0.01]	0.03 [−0.11, 0.17]
Detachment	−0.13 [−0.27, 0.00]	−0.17 [−0.30, −0.04]^*^	0.02 [−0.12, 0.16]
Antagonism	−0.03 [−0.17, 0.10]	0.08 [−0.06, 0.21]	0.45 [0.34, 0.56]^***^
Disinhibition	−0.01 [−0.14, 0.13]	0.03 [−0.10, 0.17]	0.20 [0.06, 0.33]^**^
Psychoticism	0.04 [−0.10, 0.17]	−0.01 [−0.15, 0.12]	0.15 [0.01, 0.28]^*^

N = 205; *p < 0.05, **p < 0.01, ***p < 0.001 (two-tailed).

A linear regression with cue-based deception detectability as the criterion and the Dark Triad traits as predictors produced a statistically non-significant model [adjusted *R*^2^ = −0.01, *F*(3, 201) = 0.23, *p* = 0.88]. A hierarchical multiple regression analysis with cue-based deception detectability as the criterion variable, the PID-5 traits entered in Step 1, was stopped after Step 1 [adjusted *R*^2^ = 0.02, *F*(5, 199) = 1.77, *p* = 0.12].

### Perceived Deception Detection Ability

As seen in Table 2, among the Dark Triad traits, narcissism and psychopathy were weakly associated with perceived deception detection ability. The pattern for Machiavellianism was suggestive of a potential association. Among the PID-5 traits, Detachment was negatively associated with perceived deception detection ability.

A linear regression with perceived deception detection ability as the criterion and the Dark Triad traits as predictors produced a statistically non-significant model [adjusted *R*^2^ = 0.01, *F*(3, 201) = 2.01, *p* = 0.11]. A hierarchical multiple regression analysis with perceived deception detection ability as the criterion variable, the PID-5 traits entered in Step 1, adjusted *R*^2^ = 0.03, *F*(5, 199) = 2.42, *p* = 0.04, and the Dark Triad traits entered in Step 2, adjusted *R*^2^ = 0.03, *F*(8, 196) = 1.92, *p* = 0.06, as predictor variables was computed. In the first step, Detachment emerged as a substantial predictor [*b* = −0.30, 95% CI = (−0.55, −0.04), *p* = 0.02]. The Dark Triad traits did not account for additional variance in perceived deception detection ability above the PID-5 traits, Δ*R*^2^ = 0.00, *F*(3, 196) = 1.09, *p* = 0.35.

### Perceived Deception Production Ability

As seen in Table 2, all three Dark Triad traits were correlated with perceived deception production ability. Among the PID-5 traits, Antagonism, Disinhibition, and Psychoticism were associated with perceived deception production ability.

In a linear regression with perceived deception production ability as the criterion and the Dark Triad traits as predictors [adjusted *R*^2^ = 0.23, *F*(3, 201) = 21.43, *p* < 0.001], Machiavellianism [*b* = 0.48, 95% CI = (0.20, 0.76), *p* < 0.001] and psychopathy [*b* = 0.42, 95% CI = (0.10, 0.73), *p* = 0.01] emerged as substantial predictors. A hierarchical multiple regression analysis with perceived deception production ability as the criterion variable, the PID-5 traits entered as predictor variables in Step 1, adjusted *R*^2^ = 0.22, *F*(5, 199) = 12.46, *p* < 0.001, and the Dark Triad traits entered in Step 2, adjusted *R*^2^ = 0.25, *F*(8, 196) = 9.56, *p* < 0.001, was computed. In the first step, Antagonism emerged as a substantial predictor [*b* = 1.32, 95% CI = (0.95, 1.68), *p* < 0.001]. The Dark Triad traits accounted for additional variance in perceived deception production ability above the PID-5 traits, Δ*R*^2^ = 0.03, *F*(3, 196) = 3.84, *p* = 0.01. In Step 2, Antagonism remained a substantial predictor [*b* = 0.34, 95% CI = (0.11, 0.58), *p* = 0.004] and Machiavellianism emerged as an additional predictor [*b* = 0.23, 95% CI = (0.05, 0.41), *p* = 0.01].

## Discussion

The data indicate that the Dark Triad traits and Antagonism are associated with perceived deception production ability, but not (substantially) with perceived deception detection ability and cue-based deception detectability. This replicates the result pattern regarding the Dark Triad traits and regarding Antagonism [*r*(204) = 0.45], which is the maladaptive form of Big Five Agreeableness [*r*(1447) = −0.28; Giammarco et al., 2013]. Also, a comparable adjusted *R*^2^ = 0.23 was found for the Dark Triad traits as predictors of perceived ability to deceive [adjusted *R*^2^s = 0.12–0.22; Giammarco et al., 2013].

Beyond Antagonism and among the PID-5 maladaptive personality traits, Disinhibition and Psychoticism were associated with perceived deception production ability. Controlling for the shared variance in a regression model, only Antagonism remained a substantial predictor of perceived deception production ability. Interestingly, when the Dark Triad traits and the PID-5 traits as a whole – PID-5 Antagonism is one of the proposed origins of the shared variance of the Dark Triad – were used as predictor variables of perceived deception production ability, only Machiavellianism remained a substantial predictor among the Dark Triad traits. This result suggests that Machiavellianism can uniquely explain variance above the shared variance of the Dark Triad traits and PID-5 maladaptive personality traits in perceived deception production ability. This contrasts with a meta-analysis on the Dark Triad that suggested that “psychopathy runs the show” regarding psychosocial correlates of the Dark Triad (Muris et al., 2017), suggesting that at least in the psychosocial relevant domain of deception, Machiavellianism might play an important role. Given that Machiavellianism is associated with deception production frequency (Kashy and DePaulo, 1996; Baughman et al., 2014; Jonason et al., 2014b) and amplitude (high-stakes deception; Azizli et al., 2016), a relatively high perceived deception production ability might be a necessary precondition or a consequence (or both) of this high-frequency, high-amplitude deceptive behavioral pattern.

Detachment was negatively associated with perceived deception detection ability and suggestively with cue-based deception detectability, which might be explained partially by the central interpersonally outcome of Detachment, which is withdrawal from other people. This social withdrawal might lead individuals high in Detachment to regard their deception detection skills as being relatively low, given the relative absence of social interaction as a precondition for potential deception ability acquisition and evaluation. This finding further supports the relevance of Detachment in the context of deception, for example, previously, Detachment has been found to be negatively correlated with response bias in deception detection (Wissing and Reinhard, 2017).

All three Dark Triad traits are multi-dimensional constructs: narcissism can be differentiated in grandiose vs. vulnerable expressions (Miller and Campbell, 2008; Pincus and Lukowitsky, 2010) and is conceptualized as consisting of three higher order factors (Antagonism, Neuroticism, and Agentic Extraversion) (Miller et al., 2016); psychopathy consists of primary and secondary forms (Levenson et al., 1995); Machiavellianism can be defined as a two-dimensional construct (tactics vs. views; Monaghan et al., 2016). The used SD3 measure of the Dark Triad traits tends to capture secondary psychopathy and grandiose narcissism (Jones and Paulhus, 2014). The distinction between Machiavellianism and psychopathy of the SD3 is based on impulsivity (low for Machiavellianism, high for psychopathy; see also Jones and Paulhus (2009)), but this difference in impulsivity has been questioned (Miller et al., 2017). The conceptual distinctiveness of the Dark Triad traits has also been questioned by a meta-analysis (Muris et al., 2017). Given the uncaptured multi-dimensionality and partially questionable validity of the constructs, the results of the present study might be limited and should be resolved by future studies. Additionally, it is worth stressing that self-rated ability and actual ability are not necessarily associated and might be orthogonal or even negatively correlated.

While the usage of MTurk samples is debatable, there is evidence that they are comparable to laboratory scenarios (Thomas and Clifford, 2017). The present study was based entirely on self-report instruments, suggesting that the reported results might be subject to method bias. Effect sizes were relatively low, with the exception of self-perceived deception production ability. The used deception scales were self-constructed and not properly validated. While exploratory factor analysis, internal consistency, and general factor saturation indices suggested good scale properties – and the perceived deception production ability scale showed converged validity with the PATD scale (Schneider and Goffin, 2012) in terms of close intercorrelation point estimates with the Dark Triad traits – the results should be replicated with properly validated measures by future studies before building upon them in other ways, for example, future studies might try to replicate the result pattern of this study regarding the Dark Triad traits and Antagonism as predictors of perceived deception production ability with the PATD scale (Schneider and Goffin, 2012) as the criterion variable. Also, studies with more statistical power should be conducted to investigate the potential association between narcissism and psychopathy with perceived deception detection ability. Future studies might also explore the ecological validity of the deception perceptions – when properly validated measures exist – by testing if these are predictive of actual deceptive behavior.

## Data Availability

The datasets generated for this study are available on request to the corresponding author.

## Ethics Statement

The study was conducted in full accordance with the Ethical Guidelines of the German Association of Psychologists (DGPs) and the American Psychological Association (APA). Moreover, by the time the data were acquired, it was also not required at Kassel University, nor at most other German universities to seek ethics approval for simple studies on personality and attitudes. The study exclusively makes use of anonymous questionnaires. No identifying information was obtained from participants. The participants were explicitly informed that the data are treated confidentially. Every participant had to agree to the following statements: “I understand that my participation is voluntary and that I may withdraw from the study at any time without explanation” and “I hereby confirm that I am at least 18 years old, and that I agree to take part in this study.” Furthermore, they could withdraw from the study at any time.

## Author Contributions

All authors listed have made a substantial, direct and intellectual contribution to the work, and approved it for publication.

### Conflict of Interest Statement

The authors declare that the research was conducted in the absence of any commercial or financial relationships that could be construed as a potential conflict of interest.

## Footnotes

^1^This recoding step is necessary, because (1) perceived low and high cue-frequency/-amplitude under deception relative to no deception can make a cue deception detection relevant, and (2) without the recoding step, cues with perceived low vs. high cue-frequency/-amplitude under deception relative to no deception would cancel each other out.
